# Impact of HIV-1 infection on the IGF-1 axis and angiogenic factors in pregnant Cameroonian women receiving antiretroviral therapy

**DOI:** 10.1371/journal.pone.0215825

**Published:** 2019-05-01

**Authors:** Livo F. Esemu, Emile K. Yuosembom, Rui Fang, Shayne Rasay, Barriere A. Y. Fodjo, John T. Nguasong, Winifrida Kidima, Gabriel L. Ekali, John J. Chen, Lishomwa Ndhlovu, Jude D. Bigoga, Diane W. Taylor, Rose G. F. Leke, Anna Babakhanyan

**Affiliations:** 1 The Biotechnology Center, University of Yaoundé I, Yaoundé, Cameroon; 2 Department of Biochemistry, University of Yaoundé I, Yaoundé, Cameroon; 3 Center for Medical Research, Institute of Medical Research and Medicinal Plant Study, Yaoundé, Cameroon; 4 Biostatistics Core, Department of Tropical Medicine, Medical Microbiology and Pharmacology, University of Hawaii at Manoa, John A. Burns School of Medicine, Honolulu, Hawaii, United States of America; 5 Department of Tropical Medicine, Medical Microbiology and Pharmacology, University of Hawaii at Manoa, John A. Burns School of Medicine, Honolulu, Hawaii, United States of America; 6 Department of Zoology, College of Natural and Applied Sciences, University of Dar es Salaam, Dar es Salaam, Tanzania; Management Sciences for Health, ETHIOPIA

## Abstract

Although mother-to-child transmission of HIV has dramatically declined, the number of *in utero* HIV-exposed, uninfected infants is on the increase. HIV-exposed infants are at an increased risk of mortality, morbidity and slower early growth than their non-HIV exposed counterparts. Maternal HIV increases the risk of having preterm deliveries, intrauterine growth restriction and low birth weight babies. However, the mechanism underlying dysregulation of fetal growth in HIV-infected pregnant women is unknown. We sought to determine whether maternal HIV is associated with dysregulation of the insulin-like growth factor (IGF) axis, some angiogenic factors or other related biomarkers that regulate fetal growth. A total of 102 normotensive pregnant women were enrolled in a small cross-sectional study. Amongst these were thirty-one HIV-1 positive women receiving combination antiretroviral therapy (cART) (Mean age: 30.0 ± 5.1 years; % on ART: 83.9%; median plasma viral load: 683 copies/ml; median CD4 count: 350 cells/ul) and 71 HIV uninfected women (mean age: 27.3 ± 5.8) recruited at delivery. A panel of biomarkers including IGF1 and IGF binding proteins (IGFBP1, IGFBP3), angiopoietins (ANG) 1 and 2, matrix metalloproteinases (MMP) 2 and 9, and galectin 13, was measured in plasma collected from the placental intervillous space. The levels of IGF1, IGFBP1, ANG1, ANG2, MMP2, MMP9 and Gal-13 were not affected by maternal HIV, even when adjusted for maternal factors in linear regression models (all p>0.05). It was observed that HIV-infection in pregnancy did not significantly affect key markers of the IGF axis and angiogenic factors. If anything, it did not affect women. These findings highlight the importance of the use of ART during pregnancy, which maintains factors necessary for fetal development closer to those of healthy women. However, decrease in IGF1 levels might be exacerbated in women con-infected with HIV and malaria.

## Introduction

In sub-Saharan Africa, women disproportionately bear the burden of the HIV epidemic [1,2]. Each year, 1.4 million HIV-infected women become pregnant [1], with up to 5.3% of those pregnant being HIV positive in many African countries [3]. In Cameroon, the national HIV prevalence in 2011 was 5.6% in women and 2.9% in men, but the prevalence of HIV among pregnant women was 7.8% [3,4]. Maternal HIV-1 infection increases the risk of pre-term birth (<37 weeks of gestation), small-for-gestational age babies, and fetal intrauterine growth restriction [5–9], resulting in low birth weight (LBW) infants (<2500g) [10–12]. Low birth weight occurs in over 20 million children and 95% of this condition is observed in developing countries [13,14]. Approximately 10% of children born to HIV positive Cameroonian women under prolonged HAART were born with LBW[15]. LBW is a significant cause of infant morbidity and increases the risk of mortality during the first year of life by 40-fold [16].

Mechanisms underlying LBW among HIV-exposed infants remains unknown. In term deliveries, HIV-associated LBW is likely to be caused by several factors, but dysregulated vasculogenesis in the placenta is likely to be an important component [17]. Early events such as implantation and development of the placenta are critical for successful pregnancy outcomes[18]. Placental vascular development is tightly regulated by pro-angiogenic angiopoietin 1 (ANG1) and anti-angionetic angiopoietin 2 (ANG2) [19]. During the first trimester, angiogenesis is important for remodeling of uterine spiral arteries into low resistance, high capacity vessels [17,20], which continues until mid-second trimester [19–23]. Dysfunctional remodeling of uterine spiral arteries is associated with complications of pregnancy, such as preeclampsia[24], gestational diabetes mellitus[25], Intra Uterine Growth Restriction[26], and Small for Gestational Age in the neonate [27].

Another important regulator of placental and fetal growth is the insulin like growth factor (IGF1)[28]. During pregnancy, IGF1 and its regulatory proteins are produced by placental trophoblasts and fetal cells, with the fetal liver being the main source of IGF after birth [29]. IGF1 plays a role in trophoblast migration, invasion, differentiation as well as proliferation. It also functions to influence placental angiogenesis and therefore transplacental transfer of nutrients such as amino acids and glucose. IGF receptors are found on placental cells that mediate IGF activity[30,31]. However, placental bioavailability of IGF1 is modulated by the IGF binding proteins: IGFBP1 and IGFBP3 [32,33]. Changes in IGF1 levels in maternal, placental or fetal compartments during the first trimester have been implicated in fetal growth restriction and LBW and would likely remain altered throughout pregnancy[28,29,34,35].

In addition, galectins are expressed at the maternal-fetal interface of the placenta and play key roles in placental formation and vascularization[36]. Among the 19 galectins known, placental galectin 13 (or placental protein 13, Gal-13) has been shown to be expressed by the syncytiotrophoblast, endovascular trophoblast and decidual spiral arteries and is important in trophoblast invasion and vascular remodeling during placentation [37]. Gal-13 is also regarded as an endogenous danger/damage signal, as its secretion from the syncytiotrophoblast is dramatically upregulated at the onset of preeclampsia and the hemolysis, elevated liver enzymes and low platelet count syndrome [36]. This lectin likely also plays an important role in feto-maternal tolerance, as it has been shown to promote apoptosis of activated T cells and macrophages[36].

Finally, placental matrix metalloproteinases (MMP) are proteolytic enzymes that have been shown to have a vital role in trophoblast invasion, regulation of vascular endothelial cell functions and placental angiogenesis[38,39]. The MMP function by essentially degrading the extracellular matrix (ECM), releasing growth factors that allow the activation of signals that are important for angiogenesis [40]. For instance, MMP2 and MMP9 are secreted by placental trophoblasts and are critical in trophoblast invasion, vascular endothelial cell migration, attachment, proliferation and survival; therefore, supporting angiogenesis[39,41]. These enzymes have been described as potential candidates in the pathogenesis of preeclampsia [42]. The impact of HIV on levels of angiopoietins, MMPs, IGF1 and Gal-13 has not been investigated. Based on the importance of these factors in the homeostasis of pregnancy, it is plausible that HIV-associated immune activation[43] may dysregulate levels of angiopoietins, MMPs, IGF1 and gal-13. Thus, in this pilot study, the impact of maternal HIV infection on angiopoietins, IGF1 and IGFBPs, MMPs and gal-13 in normotensive pregnant women receiving antiretroviral therapy was examined.

## Materials and methods

### Ethical considerations

The archival, coded samples used in the current study were exempt from human subject research by the Committee on Human Studies, University of Hawaii, Manoa (CHS 22572). The original study protocol was reviewed and approved by the National Ethics Committee Cameroon (No 2013/11/366/L/CNERSH/SP) and the Institutional Review Board of the University of Hawaii (CHS 21370). Written informed consent was obtained from each woman specifying their sample would be used for further studies, prior to enrollment into the study.

### Study site and population

In this pilot cross sectional study, archival plasma samples, obtained from a study carried out between January 2014 and September 2015 at the Yaoundé Central Hospital Maternity, a referral teaching hospital in Yaoundé, Cameroon were used. The prevalence of HIV in the city of Yaoundé is 4.4% [44]. The goal of the mother study was to investigate underlying mechanisms to poorer health observed in children born to HIV-positive women. A total of 102 mother-neonate pairs were recruited in the study at delivery. Women with pre-existing health conditions [e.g. diabetes, preeclampsia and Hemolysis, Elevated Liver enzymes, Low platelet count (HELLP) syndrome] and/or had spontaneous abortions were excluded from the study. Information on each woman’s demographic and clinical history including HIV status, ART intake, use of the intermittent preventive treatment (IPT) with sulphadoxine pyrimethamine (SP) and insecticide treated bednets (ITN) during pregnancy was available. The birth weight, length and APGAR score of newborn were also available. Gestational age was estimated based on date of last menstrual period or ultrasound scan data when available. Women with axillary temperature greater than 37.5°C were considered as having fever. Neonates born between 28 and 37 weeks were classified as premature. Singletons weighing less than 2,500 grams were considered LBW babies. Women were tested for HIV during pregnancy and vaccinated with tetanus vaccine according to national guidelines. All HIV positive women were placed on cART following national guidelines.

### Sample collection

Maternal venous blood and cord blood were collected in EDTA and sodium heparin tubes while blood from intervillous space (IVS) was obtained using the pool-biopsy method[45], processed and preserved at -20°C until analyses. In addition, impression smears of placental tissue were prepared and a piece of placental biopsy was stored in 10% buffered formalin for histological studies.

#### HIV RNA levels

HIV diagnostic data was available from the medical records at Yaoundé Central Hospital. HIV copy number was determined for all HIV positive women when sufficient amount of plasma was available (n = 14). Testing was conducted at the Chantal Biya International HIV Reference Center, Yaoundé, Cameroon using the using Abbott RealTime PCR HIV-1 kit (Abbott Park, Illinois, USA). Lower detection limit of the assay was less than 150 copies/ml; upper detection limit was 10,000,000 copies/ml.

#### Diagnosis of peripheral malaria, placental malaria and anemia

*P*. *falciparum* infections in peripheral, IVS and cord blood were detected by microscopy as described previously[46]. Peripheral blood smears were microscopically examined for presence of *P*. *falciparum*, *P*. *ovale*, *P*. *malariae*, *P*. *vivax*. Placental biopsies were also fixed in buffered formalin, embedded, stained with hemotoxylin-eosin, and examined for parasites. A woman was considered to have placental malaria (PM) if infected erythrocytes were detected in blood smears of IVS, impression smears of villous tissue, or histological sections of the placenta [47]. Maternal peripheral blood was used to determine the hemoglobin levels (Hb) using HemoCue Hb 201 (HemoCue, Sweden). Anemia was defined as Hb less than 11 g/dl [48].

#### Detection of fetal blood contamination in placental blood

In order to confirm that IVS was collected without fetal blood contamination, the degree of purity of maternal blood was assessed using Fetal Cell Stain Kit (Simmler Inc, High Ridge, MO, United States, SKU: S0412-100) per manufacturer instructions. Positive control cord blood was used as reference.

#### Measurement of MMP2, MMP9, ANG1, ANG2, IGFBP1 and IGFBP3 levels in placental intervillous space plasma

These biomarkers were measured using Luminex Screening Assay kits (R&D Systems, MN). A four-plex cocktail containing ANG1, ANG2, IGFBP1 and IGFBP3 (R&D Systems, MN, Cat. LXSAHM-04) was used to screen at 1:2 dilution IVS plasma, MMP2 and MMP9 containing 2-plex cocktail (R&D Systems, MN, Cat. LXSAHM-02) was used to screen at 1:50 dilution IVS plasma. The assay was carried out according to the manufacturer’s instructions. Plates were washed using magnetic plate separator (Luminex, Austin, Texas, Cat# CN-0269-01) and a MAGPIX instrument (EMD Millipore, Billerica, MA) was used to read plates. The results were expressed as median fluorescence intensity (MFI). A standard curve was generated for each analyte to convert MFI into corresponding protein concentration. Protein concentrations were adjusted for dilution factors used for each analyte.

#### Measurement of IGF1 and Gal-13 levels in placental IVS plasma

IGF1 levels in IVS plasma were measured using Human IGF-I Immunoassay Quantikine ELISA kit (R&D Systems, MN, Cat. DG100) according to the manufacturer’s instruction. Gal-13 levels in IVS plasma were measured using Human placenta protein13 (PP13) ELISA Kit (My Biosource, CA, Cat. MBS293460). The plates were read using microplate reader (ELISA iMARK BioRad, S#13738, JAPAN) set at 450 nm with wavelength. Results were expressed in optical density (OD) and standard curves were used to calculated protein concentrations. For IGF1, the values were multiplied by 100 (dilution factor from plasma pretreatment step). The detectable concentration range of IGF-I was 0.007 ng/ml—0.056 ng/mL and galectin 13 was 5pg/ml - 2000pg/ml.

#### Statistical analysis

Biomarker levels, demographic and clinical variables were summarized using descriptive statistics: means and standard deviations or median and interquartile range (IQR), for continuous variables such as age or parity; and frequencies and percentages for categorical variables, e.g., maternal anemia status (yes or no) and HIV-1 infection status (yes or no). Two-sample t-tests or Mann-Whitney U-tests for continuous variables, and Chi-square tests or Fisher’s exact tests for the categorical variables were used to compare women with and without HIV-1. The biomarker values were log transformed into natural logarithm scales. The effects of maternal HIV-1 infection on levels of each of biomarkers were evaluated through linear regression models, controlling for the selected demographic and clinical variables. All p values less than 0.05 were considered significant. All statistical analysis was performed using SAS 9.4 and GraphPad Prism 7.0.

## Results

### Participant characteristics

Demographic and clinical characteristics of study participants at delivery are summarized in Table 1 and S1 Table. Overall, 102 women were enrolled in the study, 31 HIV-1 positive and 71 HIV-1 negative. HIV-1 positive and negative women were similar with respect to maternal factors: IPT use, hemoglobin level, temperature, blood pressure, peripheral malaria status, parity and pregnancy outcomes: length of gestation, proportion of singleton deliveries and C-section, neonate sex, neonate weight and prevalence of LBW babies (all p-values>0.05). However, HIV-1 positive women were older compared to their healthy counterparts (p = 0.027) with average age of 30.0 ± 5.1 vs. 27.3 ± 5.8 years, respectively. Majority (83.9%) of the HIV-1 positive pregnant women were receiving ART, and most of the women were on Tenofovir Lamivudine and Efavirenz tritherapy. HIV viral load was available for 14(47%) HIV-1 positive women with median (25th, 75th) of 683 (0, 130,680) copies/μl. CD4 counts were available for 9 (30%) HIV-1 positive women; median (25th, 75th) of 350 (248, 675) cells/μl. Four (13%) HIV-1 positive pregnant women were also infected with placental malaria.

**Table 1:** Demographic and clinical characteristics of mothers.

**Characteristic**	**HIV-1 (-)**	**HIV-1 (+)**	**p-value**
Number of enrolled participants, n	71	31	-
Age in years, mean ± SD^θ^	27.3 ± 5.8	30.0 ± 5.1	**0.027**
Maternal fever, n (%)^Φ^	18 (25.4)	6 (19.4)	0.35
Maternal weight in kg, mean ± SD^θ^	75.7 ± 12.5	73.9 ± 12.9	0.64
Maternal BMI in kg/m^2, mean ± SD^θ^	29.1 ± 4.3	28.6 ± 3.7	0.72
Maternal hemoglobin level in g/dL, mean ± SD^θ^	12.1 ± 1.6	11.7 ± 1.7	0.41
Maternal anemia, n (%)^Φ^	13 (18.3)	6 (19.4)	0.73
ART use by pregnant women, n (%)	0	26 (83.9)	-
Maternal viral load, median, (25^th^, 75^th^)	0	683 (0, 130,680)	-
Maternal CD4 Count median, (25^th^, 75^th^)	N/A	350 (248,675)	-
Maternal IPT use, n (%)^Φ^	60 (84.5)	30 (96.8)	0.18
Number of SP doses pregnant women took, median, (25^th^, 75^th^)^π^	2 (1, 3)	2 (2, 2)	0.92
Maternal bednet use, n (%)^Φ^	52 (73.2)	26 (83.9)	0.41
Maternal heart rate in beats per minute, mean ± SD^θ^	84.7 ± 13.9	88.5 ± 15.9	0.39
Maternal blood pressure in mmHg, mean ± SD^θ^			
Systolic	120.9 ± 17.6	119.7 ± 8.8	0.70
Diastolic	75.2 ± 13.3	76.1 ± 8.7	0.74
Maternal peripheral malaria by blood smears, n (%)^Φ^	11 (15.5)	4 (12.9)	0.75
Maternal parasite density in peripheral blood^£^ in parasites/uL, median (25^th^, 75^th^)^π^	1,880 (400, 15,940)	1,080 (440, 12,490)	0.61
**Malaria by RDT on maternal peripheral blood**^θ^	14 (19.7)	4 (12.9)	0.44
Placental malaria, n (%)^Φ^	10 (14.1)	3 (9.7)	0.33
Parasitemia^£^ in %, median (25^th^, 75^th^)^π^	5.35 (0.06, 26.0)	0.23 (0.03, 0.61)	0.11
Parity including current child, median (25^th^,75^th^)^π^	2 (1, 3)	3 (1, 4)	0.40
Primigravidae, n (%)^Φ^	12 (16.9)	4 (12.9)	0.46
Multigravidae, n (%)^Φ^	41 (57.8)	22 (71.0)	0.46
Length of gestation in weeks, mean ± SD^θ^	39.2 ± 3.0	38.9 ± 2.6	0.66
Preterm deliveries, n (%)^Φ^	10 (14.1)	6 (19.4)	0.59
C-section, n (%)^Φ^	6 (8.5)	5 (16.1)	0.28

The data were summarized based on the non-missing values. The total % is not 100 due to missing values or values rounded. £ Calculated for only smear positive individuals. P-values were based on

^θ^ two-sample T-tests

^π^ Mann Whitney’s tests

^Φ^ Fisher’s exact tests.

### Validation of intervillous space blood collection and placental histopathology

A total of 9 random intervillous blood samples were tested for fetal blood contamination. The average proportion of fetal erythrocytes in intervillous space blood was 1.7 ± 0.3%, which shows that the level of contamination was extremely low (S1 Fig). Thus, the sample collection methodology was validated and the experiment results are reflective of what occurs on the maternal side of the placenta.

**Fig 1:**
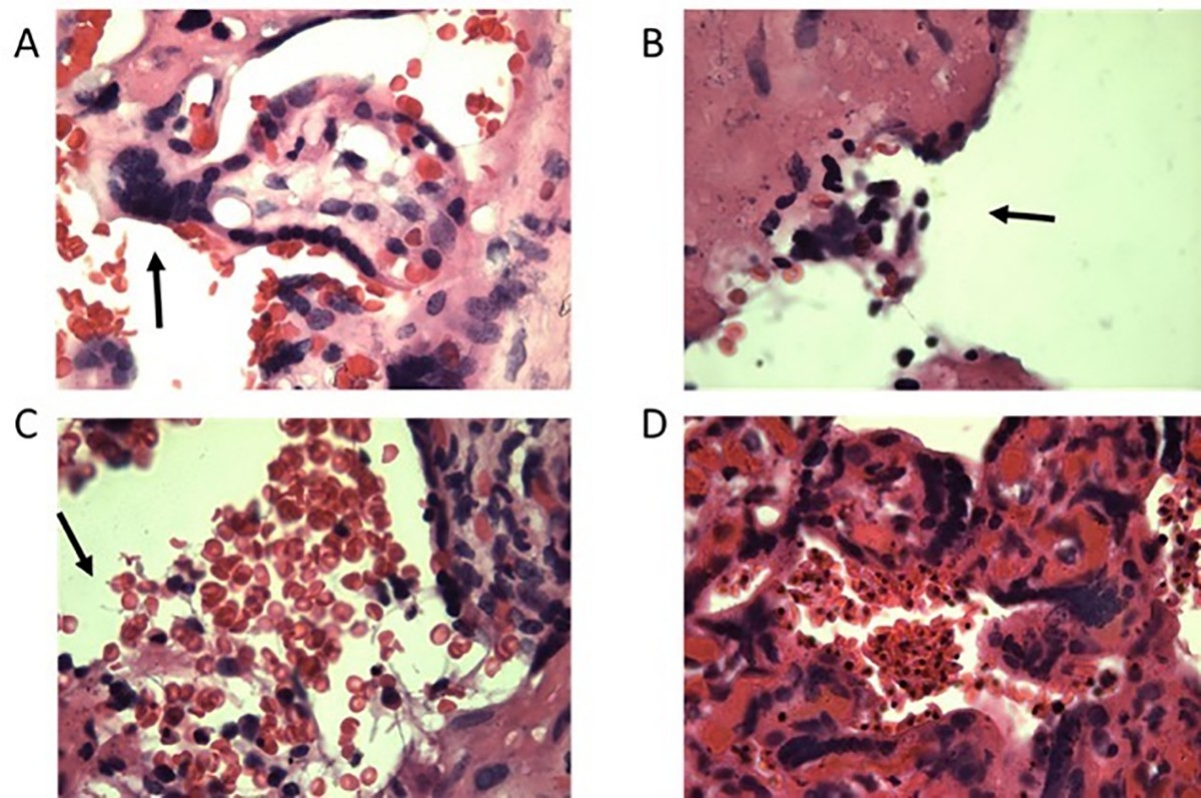
Placental histology. **(**A) HIV-1 infected woman. Arrow points to a syncytial knot. (B) HIV-1 and placenta malaria co-infected woman. Arrow points to a lesion. (C) HIV-1 and PM co-infected woman. Arrow points to fibrinoid tissue. (D) HIV-1 negative placenta malaria-positive woman. Infected erythrocytes are present in large numbers, no monocytes. 400x magnification.

Placental weight was not significantly different between HIV-1 positive and HIV negative women (p = 0.85, Table 2). In placentas from HIV-1 positive mothers, lesions and syncytial knots were occasionally observed; placentas from HIV-1 and PM co-infected women had lesions, fibrinoid tissue (Fig 1). Prevalence of placental malaria was not significantly different between HIV-1 positive (9.7%) and uninfected (14.1%) women (p = 0.33, Table 1). Except in women coinfected with malaria and HIV, women with PM did not have placental inflammation (Fig 1).

**Table 2:** Demographic and clinical characteristics of neonates.

**Characteristic**	**HIV-1 (-)**	**HIV-1 (+)**	**p-value**
Singleton deliveries, n (%)^Φ^	67 (94.4)	29 (93.6)	0.59
Male neonates, n (%)^Φ^	38 (53.5)	20 (64.5)	0.38
Placental weight in g, mean ± SD^θ^	616 ± 155	609 ± 177	0.85
Neonate weight in g, mean ± SD^θ^	3169 ± 587	3127 ± 497	0.74
Low birth weight, n (%)^Φ^	6 (8.5)	2 (6.5)	1.00
APGAR at 1min, mean ± SD^θ^	7.9 ± 1.5	8.4 ± 1.0	0.17
APGAR at 5min, mean ± SD^θ^	8.8 ± 1.4	8.9 ± 1.0	0.81
Cord malaria infection by blood smears, n (%)	0	0	-

The data were summarized based on the non-missing values. The total % is not 100 due to missing values or values rounded. £ Calculated for only smear positive individuals. P-values were based on

^θ^ two-sample T-tests

^π^ Mann Whitney tests

^Φ^ Fisher’s exact tests.

### Angiopoetin 1 and 2 are not dysregulated in HIV-1 positive women on antiretroviral therapy

The placental levels of ANG1 and ANG2 biomarkers in natural logarithm scales by HIV-1 status are presented in Table 3. There was no significant difference between HIV-1 positive and HIV-1 negative women in ANG1 (p = 0.68) and ANG2 (p = 0.20) as depicted on Table 3. In general linear regression models adjusted for maternal age and malaria status (Table 4), HIV-1 infection did not have significant impact on ANG1 (p = 0.93) and ANG2 (p = 0.33).

**Table 3:** Placental biomarker levels by HIV-1 status.

*Biomarker	HIV-1 (-) n = 71	HIV-1 (+) n = 31	p-value
ANG1(pg/ul)	10.64 ± 0.53	10.6 ± 0.52	0.68
ANG2(pg/ul)	9.22 ± 0.42	9.38 ± 0.54	0.20
IGF1 (ng/ul)	4.31 ± 0.19	4.29 ± 0.24	0.76
IGFBP1(ug/ul)	12.02 ± 0.36	12.01 ± 0.36	0.92
MMP2(ug/ul)	12.45 ± 0.30	12.60 ± 0.36	**0.066**
MMP9(ug/ul)	13.18 ± 1.05	13.15 ± 0.87	0.91
Gal-13(ug/ul)	5.70 ± 0.47	5.45 ± 0.31	**0.06**

*Biomarker levels were log transformed and the data were summarized by mean ± SD, based on non-missing values. P-values were based on two-sample T-tests.

**Table 4:** Placental biomarker level reduction due to HIV-1.

		HIV-1 (+) vs. HIV-1 (-)		Malaria (+) vs. Malaria (-)		Age	
Biomarker	R^2^	Estimate (95% CI)	p-value	Estimate (95% CI)	p-value	Estimate (95% CI)	p-value
ANG1(pg/ul)	0.081	0.012 (-0.26, 0.29)	0.93	-0.22 (-0.55, 0.11)	0.19	-0.020 (-0.043, 0.004)	0.11
ANG2(pg/ul)	0.036	0.14 (-0.14, 0.41)	0.33	-0.038 (-0.37, 0.29)	0.82	0.011 (-0.015, 0.031)	0.49
IGF1(ng/ul)	0.27	-0.086 (-0.19, 0.022)	0.12	-0.19 (-0.32, -0.067)	**0.0038**	-0.004 (-0.014, 0.006)	0.44
IGFBP1(ug/ul)	0.0038	0.10 (-0.23, 0.19)	0.84	0.12 (-0.29, 0.21)	0.75	-0.002 (-0.019, 0.016)	0.84
MMP2(ug/ul)	0.039	0.12 (-0.042, 0.28)	0.15	0.092 (-0.21, 0.15)	0.74	0.007 (-0.012, 0.016)	0.79
MMP9(ug/ul)	0.029	0.018 (-0.48, 0.52)	0.95	0.28 (-0.19, 0.93)	0.20	0.021 (-0.055, 0.028)	0.53
Gal-13(ug/ul)	0.089	-0.25 (-0.55, 0.042)	0.090	0.17 (-0.33, 0.30)	0.82	0.013 (-0.031, 0.023)	0.79

The Placental biomarker levels were in natural logarithm scales and the model was adjusted for mat maternal age and malaria status. The malaria status was confirmed by either placental malaria or maternal peripheral blood RDT. P-values were based on linear regression analyses.

**Fig 2:**
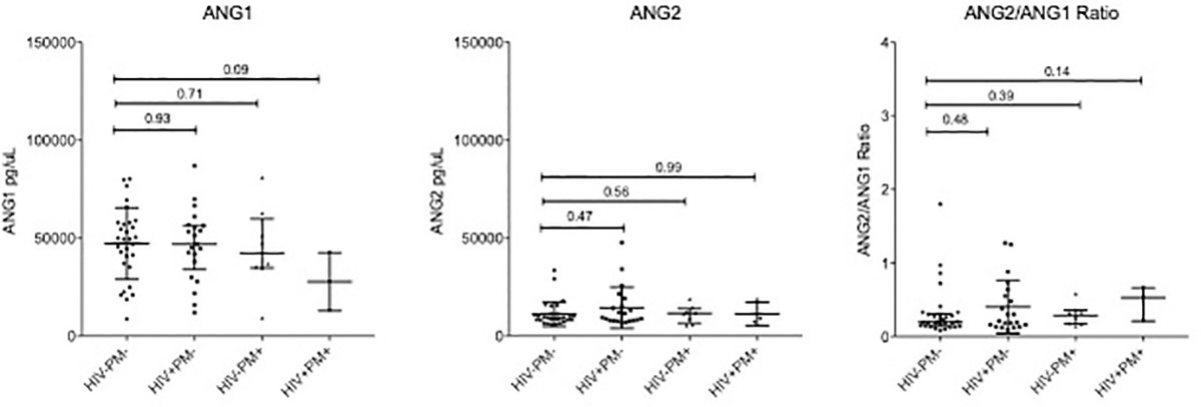
Angiopoietin levels in placental intervillous space. ANG1 and ANG2 levels, as well as ANG2/ANG1 ratio was measured in placental intervillous space in healthy (HIV-&PM-, n = 30), HIV-infected (HIV+&PM-, n = 21), PM-positive (HIV&PM+, n = 8) and co-infected (HIV+&PM+, n = 3) women. Median and interquartile ranges (IQR) are plotted; differences between the healthy and infected women were assessed using Mann-Whitney test. HIV: Human Immunodeficiency Virus; ANG1: Angiopoetin 1; ANG2: Angiopoetin 2; PM: Placenta Malaria positive mothers.

In order to determine whether angiopoetins are dysregulated during HIV-1 infection, ANG1, ANG2, as well as ANG2/ANG1 ratio were measured in placental intervillous space plasma from HIV-1 positive PM-negative and HIV-1 negative PM-negative women. No significant differences in ANG1 or ANG2 (all p>0.05) were observed between women with HIV-1 and their healthy counterparts (Fig 2). No significant differences between HIV-1 negative PM-positive women and their healthy counterparts were observed for either ANG1 or ANG2 (all p>0.05, Fig 2). ANG1 was lower in 3 co-infected pregnant women compared to healthy women, but the difference was not statistically significant (p = 0.08, Fig 2).

### IGF axis is not dysregulated in HIV-1 infected women receiving antiretroviral therapy

The placental biomarkers levels of IGF axis in natural logarithm scales by HIV-1 status are presented in Table 3. There was no significant difference between HIV-1 positive and HIV-1 negative women in IGF1 (p = 0.76) and IFGBP1 (p = 0.92). In linear regression models adjusted for maternal age and malaria status (Table 4), IGF-1 was not significantly reduced as a result of HIV-1 (p = 0.12) but due to malaria status (p = 0.0038), while no effect of HIV-1 on IFGBP1 was identified (p = 0.84).

**Fig 3:**
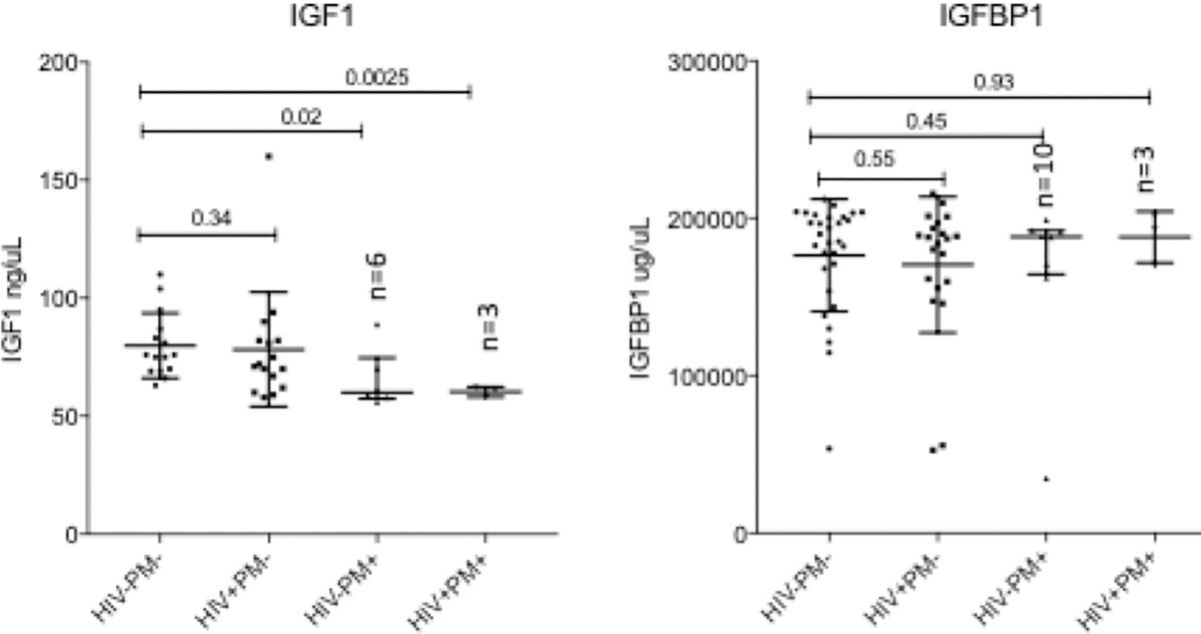
IGF1 and IGFBP1 levels in placental intervillous space. IGF1 levels were measured in placental intervillous space plasma of healthy (HIV-&PM-, n = 15), HIV-infected (HIV+&PM-, n = 16), PM-positive (HIV-&PM+, n = 6) and co-infected (HIV+&PM+, n = 3) women. IGFBP1 and IGFBP3 levels were also measured in healthy (HIV-&PM-, n = 30), HIV-infected (HIV+&PM-, n = 21), PM-positive (HIV-&PM+, n = 8) and co-infected (HIV+&PM+, n = 3) women. Median and interquartile ranges (IQR) are plotted; differences between the healthy and infected women were assessed using Mann-Whitney test. HIV: Human Immunodeficiency Virus; IGF1: Insulin Growth Factor 1; IGFBP1: IGF Binding Protein 1; PM: Placenta Malaria positive mothers.

The impact of HIV-1 on IGF axis was evaluated by probing placental intervillous space plasma obtained from HIV-1 positive PM-negative, HIV-1 negative PM-negative and HIV-1 negative PM-positive women for IGF-1 and IGFBP1 and IGFBP3. Lower but not significant levels (p = 0.3) of IGF-1 were observed in HIV-1 positive PM-negative women compared to healthy women (Fig 3). No significant differences in IGFBP1 were observed between HIV-1 infected and healthy women. In linear regression models adjusted for maternal age and anemia status no significant effect of HIV-1 on IGFBP1 was identified (Table 4). IGFBP3 was not detected in any of the samples and thus excluded from analysis.

### HIV-1 is not associated with decreased levels of MMP2, MMP9 and Gal-13

The placental levels of MMP2, MMP9 and Gal-13 biomarkers in natural logarithm scales by HIV-1 status are also presented in Table 3. There was no significant difference between HIV-1 positive and HIV-1 negative women in MMP9 (p = 0.91), but marginally significant in MMP2 (p = 0.066) and Gal-13 (p = 0.060). After adjusting for maternal age and malaria status, HIV-1 status had no significant impact on MMP2, MMP9 and Gal-13 (all p>0.05, Table 4).

**Fig 4:**
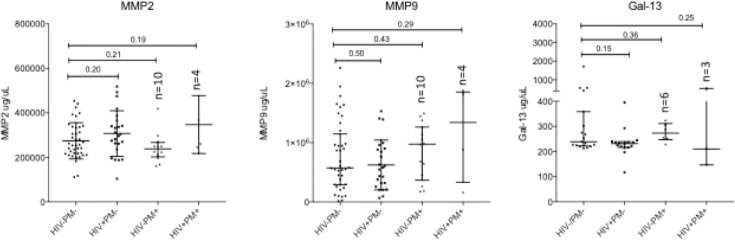
MMP and Gal 13 levels in plasma from placental intervillous space plasma. MMP2 and MMP9 levels were measured in placental intervillous space in uninfected (HIV-&PM-, n = 40), HIV-infected (HIV+&PM-, n = 24), PM-positive (HIV-&PM+, n = 12) women and co-infected (HIV+&PM+, n = 4) women. Gal-13 were measured in intervillous space plasma of healthy (HIV-&PM-, n = 17), HIV-infected (HIV+&PM-, n = 16), PM-positive (HIV-&PM+, n = 6) and co-infected (HIV+&PM+, n = 3) women. Median and interquartile ranges (IQR) are plotted; differences between the healthy and infected women were assessed using Mann-Whitney test. HIV: Human Immunodeficiency Virus; MMP: Matrix Metalloproteinase; Gal-13: Galectin-13; PM: Placenta Malaria positive mothers.

The impact of HIV-1 on additional biomarkers of placental formation and vascularization were also explored, including MMP2, MMP9 and Gal-13. No significant differences were observed between HIV-1 positive PM-negative and healthy women for MMP2 and MMP9 levels in intervillous space plasma (all p>0.05, Fig 4 and Table 3). No significant differences in MMP2 and MMP9 levels were observed between HIV-1 negative PM-positive women and their healthy counterparts (all p>0.05, Fig 4). Intervillous space plasma Gal-13 levels were not significantly different between HIV-1 positive and healthy women (Fig 4), and linear regression model showed HIV-1 had no significant impact on Gal-13 levels (Table 4).

## Discussion

The goal of our study was to determine whether maternal HIV is associated with the dysregulation of insulin-like growth factor (IGF) axis, angiogenic factors—or other related biomarkers that regulate fetal growth. In this pilot study, a panel of biomarkers implicated in placental homeostasis and fetal growth were assessed in intervillous space plasma of HIV-1 positive normotensive women on antiretroviral therapy and their HIV-1 negative counterparts. This panel of biomarkers included those involved in angiogenesis, IGF axis, as well as profile of MMPs and Gal-13. Angiogenic factors were not affected by maternal HIV-1 in our cohort of pregnant women receiving antiretroviral therapy. Angiopoetins 1 and 2 levels were not significantly different between HIV-1 positive and healthy women, even after adjusting for maternal factors.

Studies have shown that persistent HIV infection contributes to the development of chronic arterial injury and subsequent endothelial damage, atherosclerosis and thrombosis [49]. In addition, HIV-infected children have arterial stiffness and endothelial dysfunction in the absence of cardiovascular risk factors [50]. Since most of the women in our study were on ART, it is likely that combination antiretroviral therapy (cART) prevents angiopoietin dysregulation. Graham et al. reported that in non-pregnant Kenyan HIV-1 positive women with advanced HIV infection, initiation of cART significantly lowered ANG2 levels, while ANG1 was increased [51]. In agreement with previous studies, we confirmed that HIV-1 negative women with PM had significantly lower ANG1 levels compared to uninfected pregnant women [52,53]. The reason for this observation may stem from malaria parasite level in blood of PM+ women. Silver et al, (2010) found an inverse association between parasitemia and ANG1 levels [54]. With very low parasitemia in malaria positive women (0.13%) in this study, there is possibly no major alteration on the levels ANG1. This might explain the minimal changes observed between PM positive women when compared to HIV-1 negative, PM negative women.

MMP2 and MMP9 levels in intervillous space plasma were not significantly different between HIV-1 positive and healthy pregnant women. MMPs are involved in vascular remodeling and vasculogenesis, especially in new blood vessel formation and angiogenesis [39]. In line with findings for angiopoietins, these data support the fact that in HIV-1 infected pregnant women on antiretroviral therapy, angiopoietin pathway is not dysregulated. Placental malaria did not have any significant effect on MMP2 or MMP9 in this study. This is in line with a previous study, in which no significant changes in plasma levels of MMP9 were observed in children with malaria infection compared to malaria negative children[55].

In linear regression models, maternal HIV-1 did not significantly associate with lower IGF1 in placental intervillous space plasma. Lower IGF1 levels were observed in HIV-infected Ugandan children [56]. In non-pregnant adults, however, serum IGF1 has been shown to depend on level of immunodeficiency in HIV-infection and it was significantly higher in patients treated with protease inhibitors-based regimen compared to non-nucleoside reverse transcriptase inhibitors and healthy subjects [57]. Similar findings were also reported by Matarazzo et al, who found an association between decreased IGF1 levels and diseases progression in HIV-1 positive individuals [58]. In this study, IGF1 was significantly higher in HIV-1 negative PM- positive women when compared to their healthy counterparts as previously described [59]. Further, in a small subset of HIV-1 positive/PM+ co-infected Cameroonian pregnant women in this study, IGF1 levels were significantly lower compared to healthy pregnant women, indicating that HIV-1 further exacerbates PM- associated dysregulation of IGF axis.

Gal-13 is critical in trophoblast invasion during placentation and has also been reported to have angiogenic effects in the placenta [60]. Studies in animal model show that the expression of Gal-13 increases vasodilation [60] and therefore placental perfusion. In addition, lack of expression of Gal-13 has been shown to impair syncytialization [61] and hence subsequent placental hormone production by syncytiotrophoblast, which is vital in the development of the placenta. Gal-13 levels have not been studied in HIV-1 positive pregnant women, but they have been described for other pathological pregnancy conditions. No significant difference was found in Gal-13 intervillous space plasma levels between HIV-1 women under cART and their uninfected counterparts. Studies by Than et al. showed that Gal-13 placental expression was lower in preterm preeclamptic placentas compared to preterm control placentas [62], while maternal peripheral serum Gal-13 concentration was higher in preterm preeclamtic women compared to preterm controls. In contrast, Sammar et al. did not observe any significant differences in maternal peripheral plasma Gal-13 levels in HIV-1 uninfected pregnant women with preeclampsia or hemolysis, elevated liver enzymes and low platelet count syndrome compared to HIV uninfected [63]. Also, there was no difference in placental Gal-13 levels between HIV-1 negative PM-positive pregnant Cameroonian women and healthy pregnant women.

This study has a number of limitations, including limited number of LBW neonates in both HIV-positive and HIV-negative groups, inability to differentiate effects of HIV from those of ART because ART is standard of care. Of course, an ideal study design would be to investigate these biomarkers with and without cART in a case-control study, it is not ethical to withhold cART from women. However, we observed that even though women have HIV, cART therapy was effective in maintaining their ANG levels close to that of HIV-negative women. A few women were not on cART or had high viral load for some reason beyond our grasp. It is worth mentioning we did not have viral load for all samples and thus could not perfectly do the analysis. Moreover, the findings from this pilot study will need to be confirmed in a larger study due to small samples size. Within the sphere of our study, Cameroon was transitioning from PMTCT Option A to Option B+. Most studies have associated infant growth with Protease Inhibitors [64,65] and very few studies have associated dysregulated fetal growth with prolonged cART. However, our sample size was a limiting factor in the assessment of this hypothesis [64,65]. The cross-sectional study design did not allow monitoring of fetal growth rate and concomitant biomarker levels over time during pregnancy. Given that many of the aforementioned factors are important for placentation, vasculogenesis and placental perfusion and fetal growth, a longitudinal study design would be more effective. At the same time, it is well established that maternal peripheral plasma levels and placental plasma levels may not be the same [53].

While a limitation of the study were the small number of cases, the study demonstrates that maternal HIV-1 infection might not have a dramatic influence on placental IGF1, IGFBP1, MMP2, MMP9, ANG1, ANG2 and Gal-13 levels in Cameroonian pregnant normotensive women with majority receiving cART. It is not clear whether inflammatory cytokines in the placental environment of HIV-1 infected mothers [66–69] or direct effect of HIV infection on syncytial trophoblasts lead to subtle dysregulation of IGF1, IGFBP1, MMP2, MMP9, ANG1, ANG2 and Gal-13 expression in the placenta [70]. Larger prospective longitudinal studies are required to determine, whether there is significant maternal HIV-associated dysregulation in the IGF1 axis and angiogenic factors during pregnancy, especially in women with low CD4 counts, and its effects on the neonate birth weight.

## Supporting information

S1 Fig Fetal blood contamination of intervillous space blood. Nine randomly selected maternal intervillous blood samples were screened for presence of fetal erythrocytes (experimental). In addition, known amount of cord blood was mixed with corresponding maternal intervillous space blood as a positive control. Percentage of fetal erythrocytes in each intervillous blood sample was determined; mean and standard deviation for the samples are presented in the figure.
(TIF)

S1 Table Other demographic and clinical characteristics of mothers. The data were summarized based on the non-missing values. The total % is not 100 due to missing values or values rounded. £ Calculated for only smear positive individuals. P-values were based on ^θ^ two-sample T-tests, ^Φ^ Fisher’s exact tests.
(DOCX)

S1 Database Database of IDCPC_Rui_Livo v3. (XLSX)
