# Supplementary figures and images for: Impact of HIV-1 infection on the IGF-1 axis and angiogenic factors in pregnant Cameroonian women receiving antiretroviral therapy
Source: PLoS One. 2019 May 1;14(5):e0215825. doi: 10.1371/journal.pone.0215825 (PMC6493724; doi:10.1371/journal.pone.0215825)

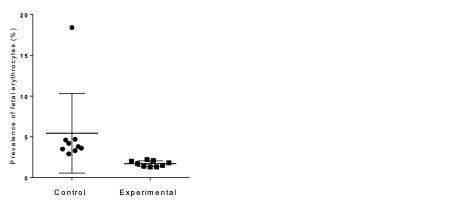

Supplement: S1 Fig — Nine randomly selected maternal intervillous blood samples were screened for presence of fetal erythrocytes (experimental). In addition, known amount of cord blood was mixed with corresponding maternal intervillous space blood as a positive control. Percentage of fetal erythrocytes in each intervillous blood sample was determined; mean and standard deviation for the samples are presented in the figure. (TIF) [file pone.0215825.s001.tif]
