# Supplementary material for: Impact of HIV-1 infection on the IGF-1 axis and angiogenic factors in pregnant Cameroonian women receiving antiretroviral therapy
Source: PLoS One. 2019 May 1;14(5):e0215825. doi: 10.1371/journal.pone.0215825 (PMC6493724; doi:10.1371/journal.pone.0215825)
Supplement: S1 Table — The data were summarized based on the non-missing values. The total % is not 100 due to missing values or values rounded. £ Calculated for only smear positive individuals. P-values were based on θ two-sample T-tests, Φ Fisher’s exact tests. (DOCX) [file pone.0215825.s002.docx]

**S1 Table. Other demographic and clinical characteristics of mothers**

| **Characteristics** | **HIV-1(-)** | **HIV-1(+)** | **p-values** |
| --- | --- | --- | --- |
| Alcohol use during pregnancy, n (%)^Φ^ | 17 (23.9) | 8 (25.8) | 0.87 |
| Smoking status during pregnancy, n (%)^Φ^ | 0 | 0 | - |
| Maternal Axillary temperature in°C, mean ± SD^θ^ | 37.4 ± 0.5 | 37.0 ± 1.1 | 0.13 |
| Height in cm, mean ± SD^θ^ | 161.0 ± 7.4 | 164.0 ± 7.4 | 0.18 |

The data were summarized based on the non-missing values. The total % is not 100 due to missing values or values rounded. £ Calculated for only smear positive individuals. P-values were based on ^θ^ two-sample T-tests, ^Φ^ Fisher’s exact tests.
